# Comparative Study of Silk-Based Magnetic Materials: Effect of Magnetic Particle Types on the Protein Structure and Biomaterial Properties

**DOI:** 10.3390/ijms21207583

**Published:** 2020-10-14

**Authors:** Ye Xue, Samuel Lofland, Xiao Hu

**Affiliations:** 1 Department of Physics and Astronomy, Rowan University, Glassboro, NJ 08028, USA; xuey5@rowan.edu (Y.X.); lofland@rowan.edu (S.L.); 2 Department of Biomedical Engineering, Rowan University, Glassboro, NJ 08028, USA; 3 Department of Molecular and Cellular Biosciences, Rowan University, Glassboro, NJ 08028, USA

**Keywords:** silk fibroin protein, composite material, magnetic nanoparticles, secondary structure, film

## Abstract

This study investigates combining the good biocompatibility and flexibility of silk protein with three types of widely used magnetic nanoparticles to comparatively explore their structures, properties and potential applications in the sustainability and biomaterial fields. The secondary structure of silk protein was quantitatively studied by infrared spectroscopy. It was found that magnetite (Fe_3_O_4_) and barium hexaferrite (BaFe_12_O_19_) can prohibit β-sheet crystal due to strong coordination bonding between Fe^3+^ ions and carboxylate ions on silk fibroin chains where cobalt particles showed minimal effect. This was confirmed by thermal analysis, where a high temperature degradation peak was found above 640 °C in both Fe_3_O_4_ and BaFe_12_O_19_ samples. This was consistent with the magnetization studies that indicated that part of the Fe in the Fe_3_O_4_ and BaFe_12_O_19_ was no longer magnetic in the composite, presumably forming new phases. All three types of magnetic composites films maintained high magnetization, showing potential applications in MRI imaging, tissue regeneration, magnetic hyperthermia and controlled drug delivery in the future.

## 1. Introduction

Magnetic nanoparticles have been widely used in the biomedical fields such as targeted drug delivery, biosensors, cancer treatment and medical imaging, due to their small size, tunable surface chemistry and controllable magnetization. Their magnetic properties mainly depend on the size, shape and particle distribution, which may be significantly different from those of their bulk counterparts. Magnetic particles can be easily functionalized with other biopolymer materials such as proteins to improve their mechanical flexibility and biocompatibility. BaFe_12_O_19_ is a hexagonal magnetoplumbite-type ferrite material [1,2], which has a remarkably high intrinsic coercivity, saturation magnetization and Curie temperature [3]. These unusual properties give it great potential for use in biological science applications. Cobalt is another broadly used magnetic material, which has stable chemical properties at room temperature, with a Curie temperature of up to 1121 °C [4]. Fe_3_O_4_ has been used in the biomedicine field recently, specifically with applications on magnetic resonance imaging, targeted drug delivery and tumor hyperthermia [5,6,7,8,9]. Magnetic Fe_3_O_4_ nanoparticles have high biocompatibility and low cytotoxicity [6,8], while their production method is simple and low-cost. Due to the existence of various free radical groups in human body fluids, the direct use of magnetic particles in the body can be largely limited and even cause harm to the human body. Therefore, a functional composite material that combines magnetic particles and biocompatible protein materials can significantly enhance the advantages of the two components and expand their scope of application.

Silk fibroin protein materials have shown excellent tensile strength, flexibility and biocompatibility [10,11,12]. Natural silk fibers are composed of sericin coating and silk fibroin proteins. Sericin as a protective gel coat wraps the silk fibroin, which can be removed by a degumming process [13]. Silk fibroin protein contains 18 amino acids, of which simple glycine (Gly), alanine (Ala) and serine (Ser) account for above 70% [14,15]. The secondary structure of silk fibroin includes β-sheets, random coils and α-helices [16,17,18], which greatly control the physical properties. For instance, the mechanical properties of silk fiber can be enhanced by a high content of β-sheet crystals [19,20]. The highly crosslinked silk fibril network structure through β-sheet crystals is believed to also cause the insolubility of the regenerated silk materials in water and many mild organic solvents [10,21]. Different types of silk materials, such as silk films, gels, particles and fibers, have shown great potential in biomedical applications [22,23,24,25], and by manipulating their secondary structure, one can control the release time and dose during targeted drug delivery [18,26]. A high content of β-sheet crystals, which can be stimulated through alcohol solutions or water annealing, also helps to improve cell adhesion and tissue growth [25,27,28]. In addition, the hydrophilic functional groups on the network composed of the protein chain and its crosslinked structure can make the material absorb water while still maintaining its shape and structure well, permitting its use for bone reconstruction, bioelectronics, and in vivo tumor models [29,30,31].

**Figure 1:**
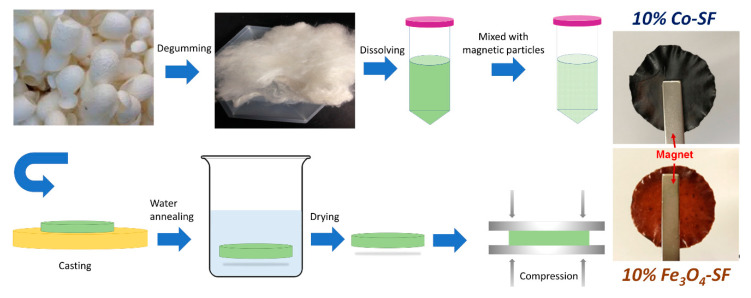
Procedures to prepare magnetic silk fibroin composite films.

In this study, three magnetic nanoparticles, M-type hexaferrite BaFe_12_O_19_ (BaM), Fe_3_O_4_, and cobalt (Co) particles were blended with silk fibroin (SF) proteins to form robust composite films (denoted as BaM-SF, Fe_3_O_4_-SF and Co-SF, respectively) by a wet-pressing method (Figure 1). Performance of the obtained silk-magnetic functional films were comparatively evaluated at various concentrations of different magnetic particles. The effect of particle concentration on the secondary structure of silk fibroins was studied by FTIR analysis. TGA and DSC were used to study the thermal stability and transitions of silk-magnetic composite films, and SEM with EDS was used to characterize the morphology of the silk films and distribution of the particles while the magnetization was studied by magnetometry. This comparative study helps us better understand the interactions between the organic matrix and the inorganic inclusions in composites, which have a variety of potential uses as sustainable or biomedical materials.

## 2. Results and Discussion

### 2.1. Structural Analysis

**Figure 2:**
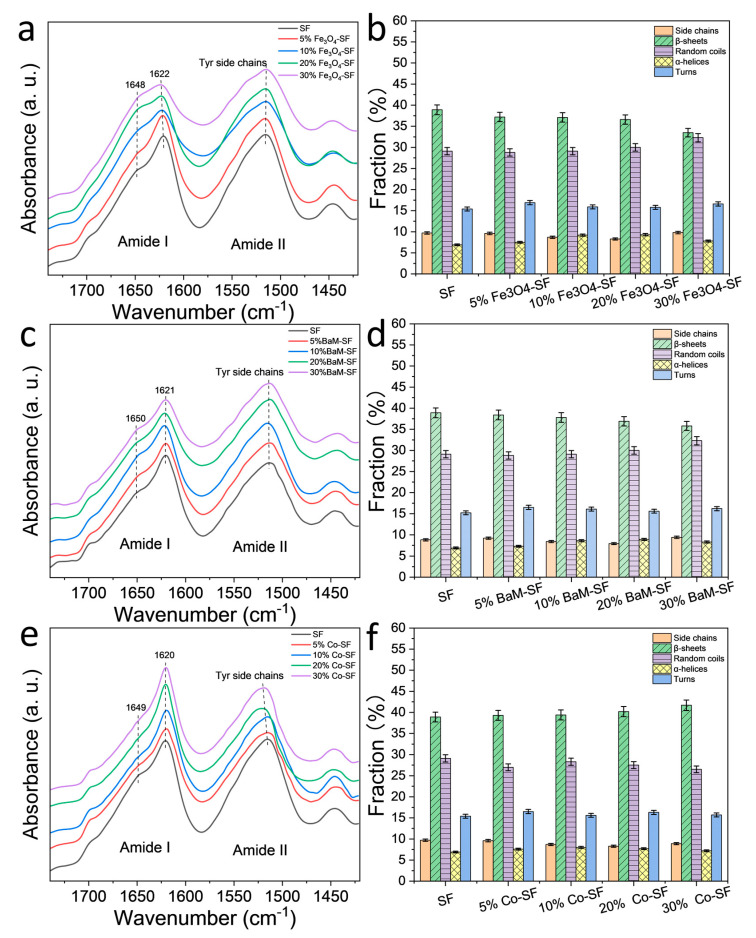
FTIR spectra of (**a**) Fe_3_O_4_-silk fibroin (SF), (**c**) BaM-SF and (**e**) Co-SF composite films. Secondary structure contents of (**b**) Fe_3_O_4_-SF, (**d**) BaM-SF and (**f**) Co-SF calculated from a Fourier self-deconvolution curve fitting method.

FTIR is an effective tool to characterize the secondary structure and functional group of silk protein materials [16,17] (Figure 2). For Fe_3_O_4_-SF samples, there is a sharp peak at 1622 cm^−1^, suggesting the β-sheet crystal structures are dominant. However, the peak shoulder at 1646 cm^−1^ (random coils structures) increased with Fe_3_O_4_ content, indicating that relative fraction of β-sheet crystals decreased. All BaM-SF samples also showed a sharp peak at 1620 cm^−1^, indicating a predominant β-sheet secondary structure due to the wet-pressing method. The peak at 1650 cm^−1^ slightly increased when more nanoparticles were present, suggesting that the BaM particles in the silk matrix can also slightly enhance the formation of α-helix or random coils structures. However, this structural change is not as significant for the Fe_3_O_4_-SF samples. Compared to the FTIR patterns of BaM-SF and Fe_3_O_4_-SF, Co-SF samples showed much sharper peaks at 1622 cm^−1^, suggesting it has the highest β-sheet crystal content among the three types of magnetic inclusions. In addition, the shoulder at 1651 cm^−1^ decreased with the increase in Co particle content. In the amide II region, all three types of composite films showed peaks around 1515 cm^−1^, which suggests Tyr side chains structure.

A quantitative analysis of the secondary structure contents was performed with a Fourier self-deconvolution (FSD) curve fitting method (Figure 2b,d,f) [16]. It shows that β-sheet content of SF sample is around 39% composed of mainly inter-molecular β-sheets [10]. The β-sheet content of the Fe_3_O_4_-SF samples decreases with Fe_3_O_4_ content, reaching 33% at 30 wt%, while the random coils content increased slightly by 29–32%. Similar behavior was found in BaM-SF samples, where the β-sheet content decreased with an increase in BaM weight fraction, while the random coils content nominally increased. In contrast, for the Co-SF samples, the secondary structure remained almost unchanged with a slight increase in β-sheet content.

### 2.2. Morphology Analysis

**Figure 3:**
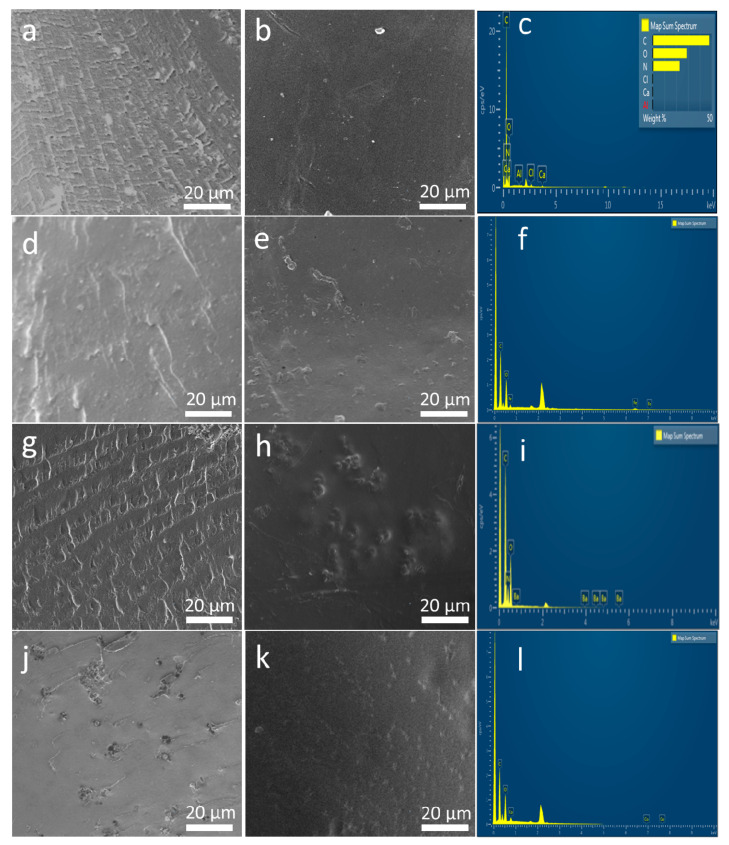
(**a**,**d**,**g**,**j**) The cross section of SF film, 20% Fe_3_O_4_-SF, 20% BaM-SF and 20% Co-SF composite, respectively; (**b**,**e**,**h**,**k**) are the surface morphology of SF film, 20% Fe_3_O_4_-SF, 20% BaM-SF and 20% Co-SF composite, respectively; (**c**,**f**,**i**,**l**) display the EDS spectra for the SF, 20% Fe_3_O_4_-SF, 20% BaM-SF and 20% Co-SF films, respectively.

Surface and cross-section morphology of SF and magnetic silk composite films are shown in Figure 3. Lamellar patterns evenly spread out across the cross section of SF and 20% BaM-SF films (Figure 3a,g). Cross section of 20% Fe_3_O_4_ (Figure 3d) showed a rougher morphology with wrinkles and densely distributed particles. Cross section of 20% Co-SF (Figure 3j) showed evenly distributed holes with connected wrinkles. When comparing the surface samples to the cross-sectional, the surface seems to be much more homogenous, containing fewer aggregates and wrinkles. SF film shows a smooth and uniform surface (Figure 3b). A 20% BaM-SF sample (Figure 3h) showed a relatively rough surface, and BaM particles distribute homogenously instead of forming big aggregates. Compared to the surface morphology of 20% BaM-SF film, 20% Fe_3_O_4_ (Figure 3e) and 20% Co-SF (Figure 3k) films showed smooth and uniform surface morphology with shallow pits. EDS spectra and analyses of the SF, 20% Fe_3_O_4_-SF, 20% BaM-SF and 20% Co-SF films are shown in Figure 3c,f,i,l, respectively. As mentioned above, most CaCl_2_ residue has been washed out of the pure SF film after the water annealing process (Figure 3c). The EDS spectra of various elements in the composites also showed evidence of Fe (6.403 keV and 0.705 keV, Figure 3f), Ba (4.465 keV and 0.779 keV, Figure 3i) and Co (6.929 keV and 0.776 keV, Figure 3l) elements in their respective composite films.

### 2.3. Thermal Analysis

**Figure 4:**
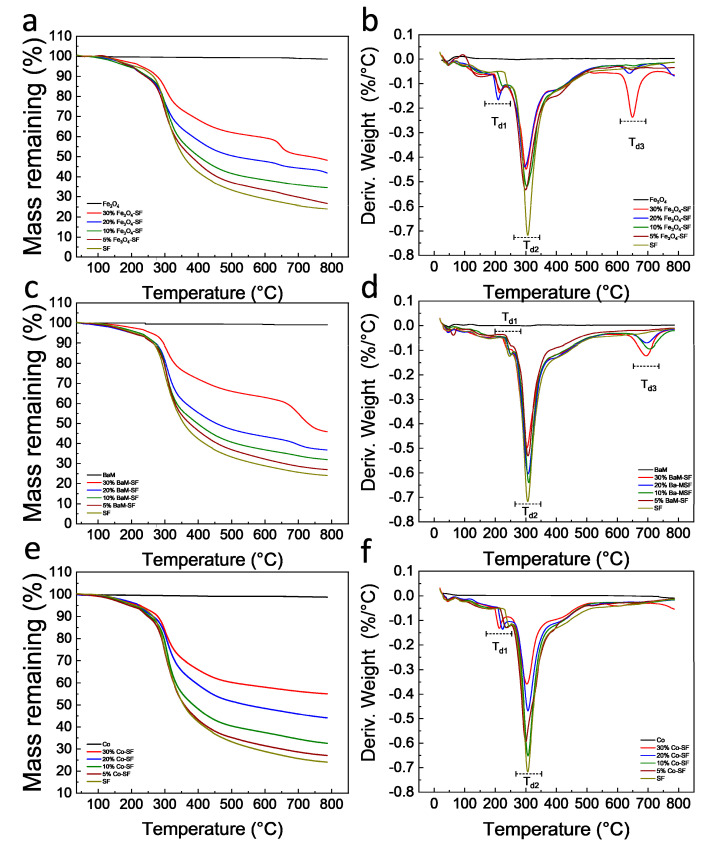
Thermogravimetric curves of (**a**) Fe_3_O_4_-SF, (**c**) BaM-SF and (**e**) Co-SF composite films. The 1st derivative TG (DTG) curves of (**b**) Fe_3_O_4_-SF, (**d**) BaM-SF and (**f**) Co-SF composite films.

Thermal stability of the silk magnetic composite films was characterized by TGA (Figure 4, Table 1). All three types of magnetic particles are thermally stable with no degradation for all of them up to 800 °C. All Fe_3_O_4_-SF composite films showed a small degradation in the range of 209~226 °C (T_d1_), a major degradation between 296 and 303 °C (T_d2_) and a third degradation around 650 °C (T_d3_). The residual weight of Fe_3_O_4_-SF samples at 800 °C was between 26.6 and 48.2%, which generally increased with Fe_3_O_4_ content. All BaM-SF composite films showed a small degradation between 238 and 251 °C (T_d1_) and a major degradation between 301 and 309 °C (T_d2_). When the BaM concentration was 10% or above, a third degradation (T_d3_) was found around 700 °C (Figure 4c,d). The residual weight of BaM-SF samples at 800 °C was between 27.1 and 46.1%, and the residual weight increased with BaM content. All Co-SF composite films showed a small degradation at 214~233 °C (T_d1_) and a major degradation at 300~307 °C (T_d2_). The residual weight of Co-SF samples at 800 °C is between 27.1 and 55.1%, and increased with Co content. However, no third degradation peak (T_d3_) was observed around 600~700 °C for any of Co-SF samples. The first small degradation is mainly from the unstable part of silk proteins [32,33,34]. BaM-SF samples showed a higher T_d1_ than that of the other two types of composites, suggesting that BaM particles were able to best protect silk materials. The third degradation for Fe_3_O_4_-SF and BaM-SF samples is probably from a stable phase that formed when the Fe combined with the silk protein.

**Figure 5:**
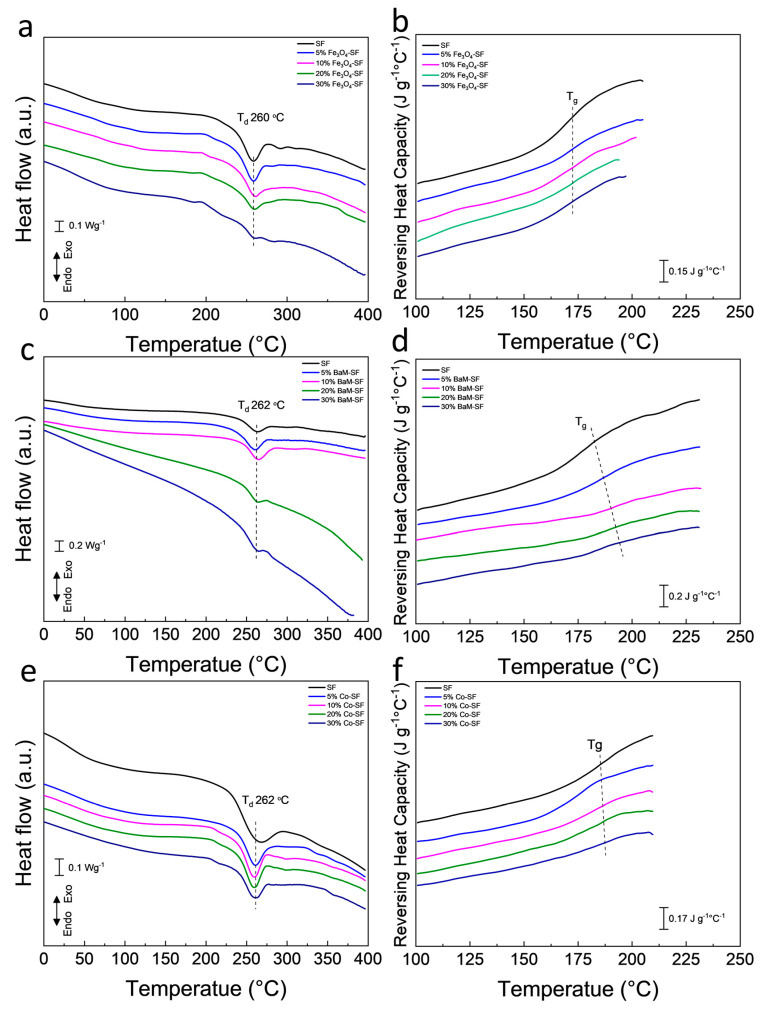
Heat flow of (**a**) Fe_3_O_4_-SF, (**c**) BaM-SF and (**e**) Co-SF composite films. Reversing heat capacity of (**b**) Fe_3_O_4_-SF, (**d**) BaM-SF and (**f**) Co-SF.

Heat flow and reversing heat capacity of magnetic silk composite films measured from DSC are shown in Figure 5. Heat flow analysis shows that all three type composite films have a major degradation about 260 °C, which is from the decomposition of silk proteins. The amorphous part of the polymer has a greater mobility with increasing temperature, which is defined as the glass transition. The glass transition is gradual and reversible, and the heat capacity of the polymer changes dramatically during this transition [35]. All Fe_3_O_4_-SF samples showed a similar glass transition temperature around 172 °C. The glass transition temperature of BaM-SF composite films increased from 172 °C for SF, to 192 °C for 30% BaM-SF, suggesting that the mobility of the amorphous structure in BaM-SF composites can be tuned by the BaM particles. All Co-SF composite films showed a glass transition temperature around 180 °C.

### 2.4. Magnetization Analysis

**Figure 6:**
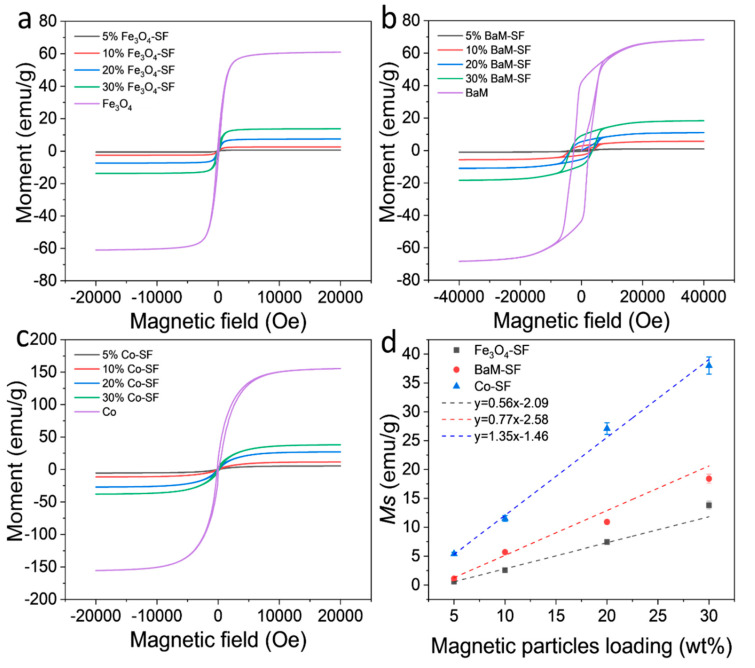
Magnetization and hysteresis loops of (**a**) Fe_3_O_4_-SF, (**b**) BaM-SF and (**c**) Co-SF composite films at room temperature. (**d**) Saturation moment of Fe_3_O_4_-SF, BaM-SF and Co-SF composite films as a function of the magnetic particle content.

Magnetization of the three types of composite films showed typical ferromagnetic behavior with coercive field of about 128 Oe (Fe_3_O_4_), 3660 Oe (BaM) and 165 Oe (Co), respectively (Figure 6a–c). The dependence of the magnetization on weight fraction of magnetic particles are shown in Figure 6d. The saturation magnetization *M_s_* of Fe_3_O_4_, BaM and Co nanoparticles is about 61 emu/g, 68 emu/g and 156 emu/g, respectively. Since silk protein matrix has magnetic susceptibility near zero, it can be assumed that the moment of the composite films depends only on the net weight of the magnetic particles. Therefore, one would anticipate that the *M_s_* value of the composite should be about *xM_s_*, where *x* is the weight fraction of magnetic particles. All samples display a linear dependence of *M_s_* on weight fraction; however, note that neither line intercepts the origin. Presumably, all three types of magnetic particles partially dissolved in the formic acid solution, saturating the solution at about 3.7 wt%, 3.4 wt% and 1.1 wt% (horizontal intercept of the graph) for Fe_3_O_4_, BaM and Co particles, respectively. These results are consistent with the study of the secondary structure of SF matrix. In any case, at sufficient loading, of three types of composite films maintain a sizable magnetization for potential use in MRI imaging or targeted drug delivery.

### 2.5. Self-Assembly Mechanism

**Figure 7:**
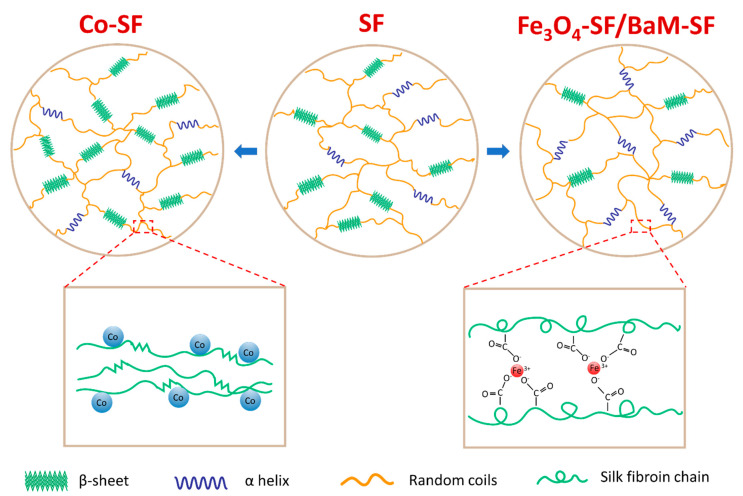
The effect of iron-based (Fe_3_O_4_ or BaM) and cobalt-based (Co) magnetic particles on the secondary structures of silk fibroin material.

With the experimental evidence and analysis provided above, we can confirm that Fe_3_O_4_ and BaM particles can slightly prevent the β-sheet crystal formation, suggesting that the Fe_3_O_4_-SF and BaM-SF composite films have more noncrystalline structures (Figure 7). This is probably caused by the strong coordination bonding between Fe^3+^ ions and carboxylate ions on silk fibroin chains [36,37,38] due to the dissolved Fe found from the magnetization studies. Most β-sheet crystals usually formed during the water annealing and wet pressing process. However, when the Fe_3_O_4_ and BaM particles were present, the strong coordination bonding limited the mobility of silk fibroin chain and further prohibited the β-sheet crystal formation (Figure 7). The unique high temperature degradation (T_d3_) of Fe_3_O_4_-SF and BaM-SF samples found in TG analysis may be another indicator of this stable phase, which is formed when Fe is combined with silk protein. On the other hand, the amount of dissolved Co is minimal as compared to that of the Fe, so the addition of Co nanoparticles has less effect on the secondary structure. Since a large amount of Co particles will occupy more space in the Co-SF film, the silk protein chains are able to be closer to each other, resulting in a slight increase in β-sheet crystals (Figure 7).

## 3. Materials and Methods

### 3.1. Materials and Synthesis

*Mori* silk cocoons were purchased from Treenway Silks (Lakewood, CO, USA). To remove the sericin coatings, silk cocoons were first degummed as reported previously [10]. An amount of 10 g of silk cocoons was added to 3 L boiling DI water with 6.36 g of NaHCO_3_ (Sigma-Aldrich, St. Louis, MO, USA). The mixture was kept boiling and stirred for 30 min. The degummed silk fibers were then removed and rinsed in DI water and the water was changed every 20 min for 3 times. The degummed silk fibroin fibers were dried in a vacuum oven overnight. Dried silk fibroin (SF) was dissolved in a formic acid solution with 4% *w*/*v* CaCl_2_ (AMRESCO Inc, Solon, OH, USA) at a concentration of 0.15 g/mL. Silk solution was firstly centrifuged to remove undissolved parts at 5000 rpm for 10 min. BaM (<100 nm, Sigma-Aldrich, St. Louis, MO, USA), Fe_3_O_4_ (50–100 nm, Alfa Aesar, Haverhill, MA, USA) and Co (~1.6 µm, Alfa Aesar, Haverhill, MA, USA) particles were added to silk solution at various weight ratios (5%, 10%, 15%, 20% and 30%). More specifically, 5% means that the weight of magnetic particles accounts for 5% of total weight of silk fibroin and magnetic particles. The mixture solution was vortexed for 10 min to disperse the nanoparticles homogeneously throughout the silk solution. The thoroughly mixed solution was cast onto a self-designed polydimethylsiloxane substrate (6-cm diameter circle) and left to dry in fume hood for 24 h (Figure 1). All samples were water annealed in DI water for 30 min. Through this procedure, silk protein chains in composite films gain additional mobility to self-assemble into a more ordered structure with high β-sheet crystallinity. In addition, the remaining formic acid and CaCl_2_ were removed during the water soaking process. To obtain aligned molecular chains with magnetic particle in the protein matrix, the just annealed wet films were also pressed under 500 kPa for 10 min (Figure 1), with a wet-pressing method [39]. Then, the wet-pressed samples were placed in a vacuum oven at 30 °C for 2 days before testing.

### 3.2. Fourier Transform Infrared Spectroscopy (FTIR)

A Bruker Tensor Fourier-transform infrared spectroscometer (FTIR, Billerica, MA, USA) was used to characterize secondary structure and functional groups of the silk-magnetic composite films. The spectrometer is equipped with a deuterated triglycine sulfate detector and a multiple reflection, horizontal MIRacle ATR attachment (with a Ge crystal, from Pike Tech. (Madison, WI, USA)). Experiments were conducted while continuously purging with nitrogen gas to eliminate unnecessary spectral contributions. The spectra were collected at a range of 4000 to 400 cm^−1^. Each run included 128 background scans and 128 sample scans at a resolution of 4 cm^−1^. Each sample was measured three times, and each run was conducted a different area or side of the same film. The ATR diamond was cleaned between samples with methanol and distilled water to remove any residue from the previous sample. Fourier self-deconvolution (FSD) curve fitting of the Amide I spectrum was conducted as reported previously [37].

### 3.3. Scanning Electron Microscopy (SEM)

The morphology was characterized with a Leo 1530 VP scanning electron microscope (SEM) (Oberkochen, Germany). To get the cross section of the composite films, samples were submerged in liquid nitrogen for 30 s each, and then were broken into small sections. All the samples were coated with gold using a desk sputter coater (Denton Vacuum LLC, Moorestown, NJ, USA) before imaging. The characterization was conducted at various magnifications with an accelerating voltage ranging between 10 and 20 kV. The elemental distribution of magnetic particles in silk matrix was also characterized by energy dispersive X-ray spectroscopy (EDS, Oxford Instruments, Abingdon, UK).

### 3.4. Thermal Property Characterization

Thermal analysis of magnetic silk composite films was conducted with simultaneous differential scanning calorimetry and thermogravimetric analysis (Q600 TA Instruments, Wilmington, DE, USA). The experiment was conducted with continuous nitrogen gas flow rate of 50 mL/min, and each specimen weighed between 5 and 10 mg. Measurements were made from 30 to 800 °C at a heating rate of 10 °C/min. Thermal transitions of all the samples were characterized by temperature-modulated differential scanning calorimetry (TMDSC). The system was purged with dry nitrogen gas at a rate of 50 mL/min and had an internal refrigerated cooling system. Indium was used to calibrate the heat flow and temperature of the DSC. Each piece of film sample weighed 5–7 mg. The measurement was conducted with Al pans at a heating rate of 2 °C/min with a modulation period of 1 min and set temperature amplitude as 0.318 K, from −40 to 400 °C.

### 3.5. Magnetic Characterization

Magnetic properties were characterized with a vibrating sample magnetometer (VSM) attachment to a Physical Property Measurement System (PPMS, Quantum Design, San Diego, CA, USA). Measurements were performed at room temperature with magnetic fields up to 4 T.

## 4. Conclusions

In this study, effects of three commonly used biocompatible magnetic particles on silk protein secondary structures and properties were discussed. Structural analysis using FTIR indicated that Fe_3_O_4_ and BaM particles prohibited β-sheet crystal formation, while the addition of Co particles had a minimal effect. The decrease in crystal structure is probably caused by the strong coordination bonding between Fe from Fe_3_O_4_/BaM and carboxylate ions on silk fibroin chains. The magnetization studies indicated that about 1.1~3.7 wt% magnetic particles were dissolved in the formic acid solution and this likely led to the formation of the phases that coincide with the high temperature degradation (>640 °C) that was seen in both Fe_3_O_4_-SF and BaM-SF composites. All three types of magnetic composite films maintained high magnetization, suggesting potential biomedical applications such as MRI imaging, tissue regeneration, magnetic hyperthermia and controlled drug delivery in the future.

## Figures and Tables

**Table 1:** Thermal properties of Fe_3_O_4_-SF, BaM-SF and Co-SF composite films *.

	T_w_ (°C)	T_d1_ (°C)	T_d2_ (°C)	T_d3_ (°C)
SF	48	245	308	-
5% Fe_3_O_4_-SF	49	219	303	657
10% Fe_3_O_4_-SF	52	226	297	659
20% Fe_3_O_4_-SF	46	209	296	641
30% Fe_3_O_4_-SF	50	215	303	650
5% BaM-SF	63	250	308	-
10% BaM-SF	64	251	309	708
20% BaM-SF	60	250	306	697
30% BaM-SF	61	238	301	696
5% Co-SF	52	233	300	-
10% Co-SF	54	234	307	-
20% Co-SF	50	223	307	-
30% Co-SF	52	214	303	-

* Data was obtained from TGA. The weight derivative peak position was used as the degradation temperature. All temperature values have an error bar within ± 0.5 °C.
